# Ruptured giant omphalocele with congenital short small intestine: a case report

**DOI:** 10.3389/fnut.2024.1421033

**Published:** 2024-07-18

**Authors:** Wenjing Zhang, Yang Wu, Cheng Pan, Xiangyun Zhang, Hong Yan, Li Zhang

**Affiliations:** ^1^Department of Pediatrics, West China Second University Hospital, Sichuan University, Chengdu, China; ^2^Key Laboratory of Birth Defects and Related Diseases of Women and Children, Sichuan University, Ministry of Education, Chengdu, China; ^3^Department of Pediatric Surgery, West China Hospital, Sichuan University, Chengdu, China; ^4^Department of Plastic, Aesthetic, Reparative and Reconstructive Surgery, West China Second University Hospital, Sichuan University, Chengdu, China

**Keywords:** omphalocele, congenital short small intestine, vacuum-sealing drainage, nutrition, perioperative management

## Abstract

We herein present a case of a ruptured giant omphalocele with congenital short small intestine. Vacuum-sealing drainage and carboxymethylcellulose silver dressing promoted wound healing after repair, avoided abdominal compartment syndrome, and reduced the risks of multiple procedures. We review the perioperative management of omphaloceles in congenital short small intestines.

## 1 Introduction

Omphalocele, which occurs in approximately 1–3 per 10,000 live births (1–3), is a midline abdominal wall defect of variable size arising at the root of the umbilical cord. The defect is covered by a capsule composed of an amnion, Wharton’s jelly, and peritoneum, which often contains herniated abdominal contents (4). A giant omphalocele (GO) is defined as a defect ≥5 cm in diameter, presence of an omphalocele, or a large omphalocele relative to the fetal abdomen (5). Capsular rupture can occur in 7–15% of children with GOs and can significantly increase the risk of complications and mortality (6, 7). The mortality rate of omphalocele with malformations is approximately seven times that of isolated omphalocele (8, 9).

A congenital short small intestine is a rare developmental malformation of the small intestine that is functionally or anatomically inadequate in length, resulting in severely reduced intestinal absorptive capacity, manifesting as vomiting, diarrhea, growth retardation, and intestinal malrotation (10, 11). Congenital short small intestine may be inherited in an autosomal recessive pattern, such as loss-of-function mutations in coxsackie and adenovirus receptor-like membrane protein, or X-linked type of inheritance (12, 13). Currently, only two studies have reported omphaloceles in congenital short bowel syndrome (7, 14), while most cases of short bowel syndrome are due to necrotizing enterocolitis and intestinal atresia (15).

Herein, we report a case of ruptured GO closed by the application of vacuum sealing drainage (VSD) combined with silver dressing and share the nutritional support strategy for congenital short small intestine.

## 2 Clinical case

**Figure 1:**
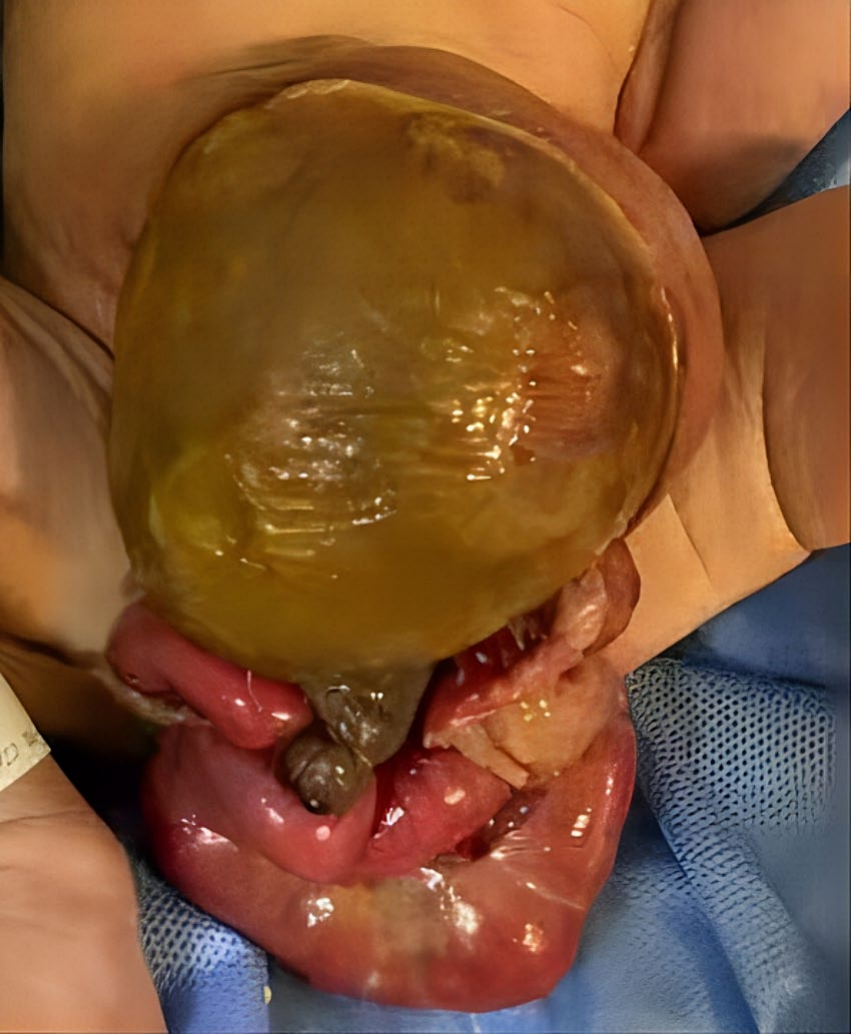
Preoperative ruptured omphalocele.

**Figure 2:**
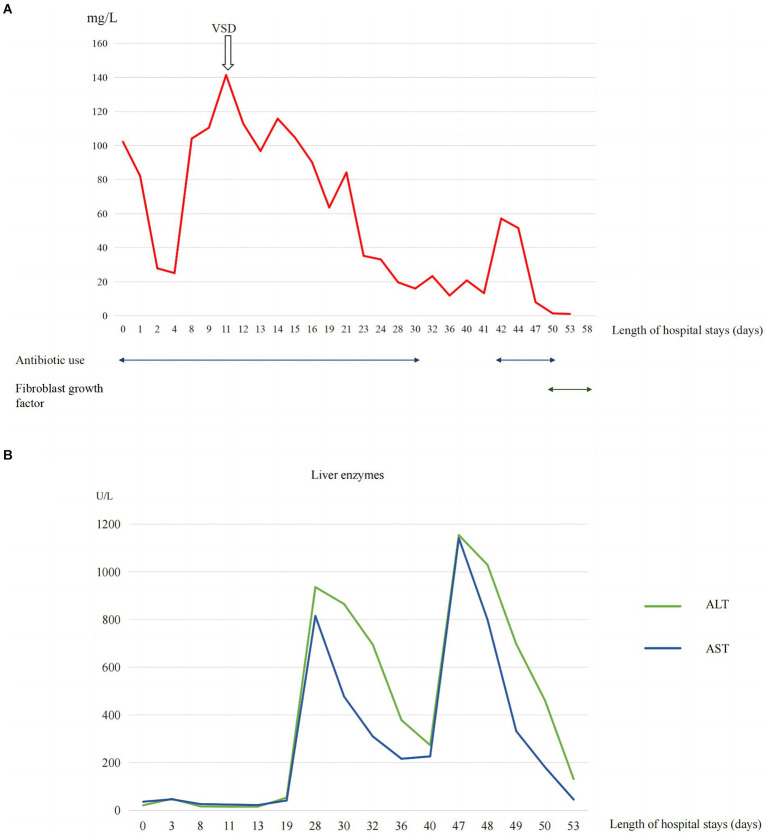
**(A)** Trend of inflammatory marker CRP levels. **(B)** Trend of liver enzyme levels.

A 2-day-old girl with a 12 × 10 cm ruptured GO was transferred to our hospital on June 5, 2023. She was born vaginally with a birth weight of 3,000 g. Gestational age was 40 + 4 weeks. Apgar scores at 1 and 5 min were 10. The bowel was partially visible through the postnatally ruptured sac (Figure 1). The neonate had dyspnea and poor peripheral perfusion on examination owing to enteral fasting and evaporative fluid loss, characterized by decreased urine output, skin mottling, and prolonged capillary refill time. Radiography revealed polydactyly of the left foot and a right-sided thoracolumbar curve. After stabilization with fluid resuscitation, vasoactive drugs, antibiotics, and mechanical ventilation, pediatric surgeons placed a silo bag that night. The silo bag was anchored to the full layer of the abdominal wall with interrupted sutures so that pressures could be added post-operatively for reduction. The membrane of the sac was completely broken. There was severe adhesion and contamination on the intestines. Insensible water loss was increased from the intestines through the postnatally ruptured sac. The entire small bowel and colon, part of the stomach, and most of the liver were in omphalocele. The herniated portion of the liver was spherical. The length of the entire small intestine was 50 cm. Postoperatively, her intra-abdominal pressure was monitored, and she had a foley catheter to drain urine, thereby reducing the increase in intra-abdominal pressure caused by urinary retention. On the 10th postoperative day, the tension in the abdominal wound increased gradually, and the inferior suture ruptured with skin erythema and exudate. Additionally, she had recurrent fever with persistently elevated C-reactive protein (CRP) levels of up to 141 mg/L (Figure 2A).

**Figure 3:**
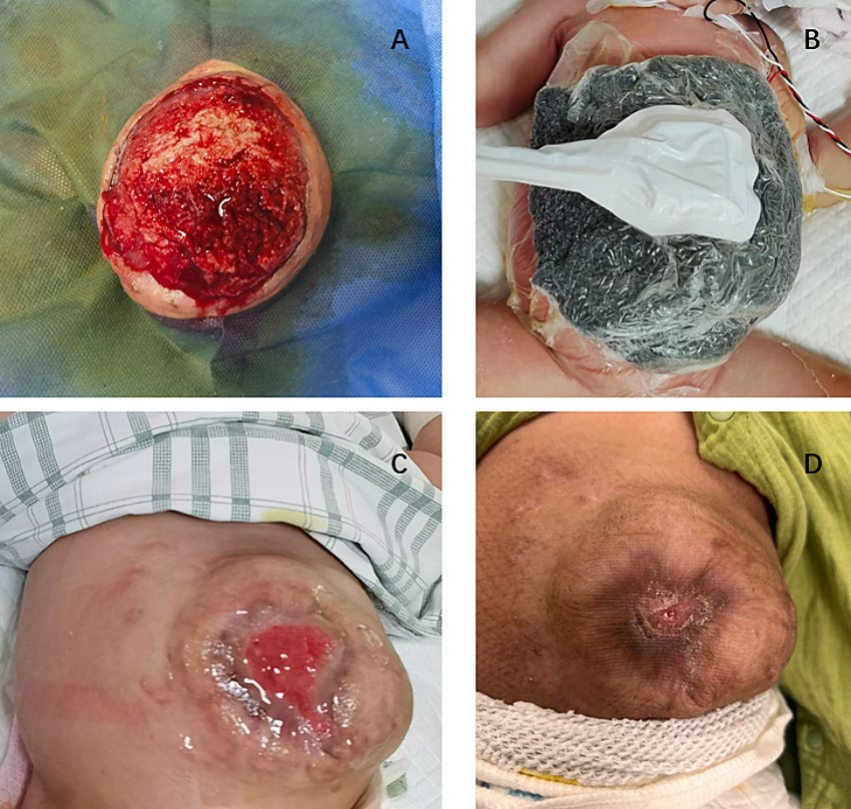
**(A)** Wound granulation tissue during replacement of VSD dressing **(B)** VSD and carboxymethylcellulose silver dressing used on the wound **(C)** Bovine basic fibroblast growth factor gel applied to the wound **(D)** Wound healing at outpatient follow-up.

Proper assessment and personalized wound repair strategy followed a multidisciplinary approach by an expert team, including neonatologists, plastic surgeons, pediatric surgeons, pediatric infectious disease specialist, enterostomal therapist, and radiologist. The protective wound material comprised three layers: the first layer was petroleum gauze, second layer was a carboxymethylcellulose silver dressing, and third layer was the closed VSD protective wound material; the VSD machine was connected. The VSD dressing was changed at the bedside 6 times over 38 days. CRP levels gradually decreased to normal without fever (Figure 2), the amount of liver exposure gradually decreased, and the wound gradually narrowed. A large amount of cellulosic exudate was observed on the surface of the antiadhesive petroleum gauze, which could be observed through the growth of granulation tissue and bled easily to touch (Figure 3), forming an intact granulation barrier. When the dressing was removed, the wound size was reduced to 2.3 × 2.6 cm; then, bovine basic fibroblast growth factor gel was applied to the wound to promote epithelialization. Finally, the abdominal wall defect was restored. On hospital days 28 and 47, alanine aminotransferase (ALT) and aspartate aminotransferase (AST) levels were markedly elevated (Figure 2B). Other liver function tests, including bilirubin, albumin, international normalized ratio, and coagulation function, were normal. ALT and AST returned to normal levels within 1 week after weaning off VSD. Full enteral feeding was achieved within 19 days after parenteral nutrition and early trophic feeding. After 11 days of mechanical ventilation, she was successfully extubated. Through 58 days of hospitalization, the infant grew from 2,930 to 4,670 g, with an average growth of 30 g per day. At the last follow-up, the infant was 6 months old and breastfed exclusively for 3 months with normal growth and development.

## 3 Discussion

Omphaloceles are frequently associated with chromosomal abnormalities or non-chromosomal syndromes. GOs have a lower incidence of chromosomal abnormalities than small-sized omphaloceles but are more likely to cause pulmonary dysplasia and often require longer mechanical ventilation (16). The mother of the patient had no relevant risk factors for omphalocele, including low or advanced maternal age, prenatal overweight, maternal smoking, or alcohol consumption (17–19). Chromosomal microarray and whole-genome sequencing of the neonate revealed no abnormalities. Eleven days of postoperative mechanical ventilation was shorter than the mean postoperative duration of mechanical ventilation reported in previous studies in children with non-isolated omphalocele (20). This may be related to the absence of severe cardiac malformations or pulmonary hypoplasia, in addition to frequent ventilator support mode (synchronized intermittent mandatory ventilation, pressure support ventilation, and volume guarantee), fluid balance, correction of hypoalbuminemia, and intra-abdominal pressure monitoring.

Staged closure avoids acute increases in intra-abdominal pressure (21). The first part of the staging surgery included protection from intestinal perfusion, which creates conditions for the establishment of enteral feeding at an early stage. Wharton’s jelly is a component of the hernia sac’s components (4). Wharton’s jelly-derived mesenchymal stem cells and fibroblasts can promote the proliferation of epithelial cells and excretion of extracellular matrix (22). Therefore, before the application of VSD, the liver covered by the sac can accelerate the growth of granulation tissue and repair wounds. In this case, the second stage was six bedside VSD dressing changes over 38 days, and the third stage was VSD evacuation using fibroblast growth factor to promote wound epithelization and healing, which avoided infection, repeat anesthesia, and transport, and shortened the duration of VSD treatment, antibiotic course, and length of hospital stay. VSD is a non-surgical closed wound method that produces negative pressure continuously or intermittently through a pump, thereby increasing blood circulation, inhibiting bacterial growth, reducing wound edema, and accelerating wound healing (23, 24). VSD is a safe, convenient, and effective treatment for GOs, with a median treatment time of 68 days, median time of 19 days for full enteral feeding, and wound healing time with combined silver dressings varying (25–27). Carboxymethylcellulose silver dressing is a sterile, non-adhesive, and non-occlusive dressing comprising silver sulfate and a polyester textile mesh impregnated with hydrocolloid particles and petrolatum. When the dressing comes in contact with the wound fluid, silver sulfate releases silver ions and promotes wound healing (28). Abdominal wall plastic surgery is expected to be performed at 2 years of age.

**Figure 4:**
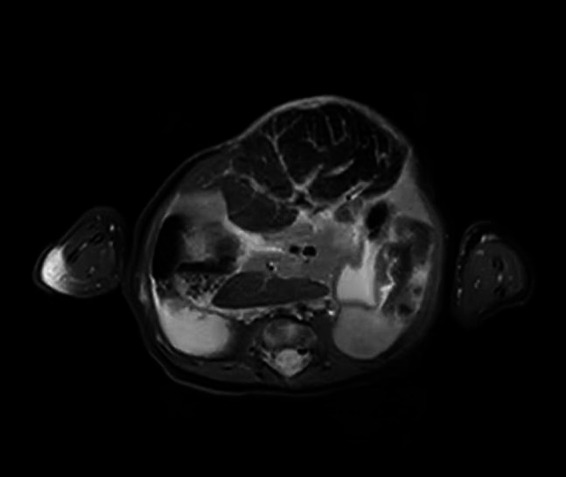
Abdominal magnetic resonance imaging.

This patient developed markedly elevated serum ALT and AST levels. These suggested that the biochemical liver alterations were caused by hepatocellular injury. The etiologies of hepatocellular injury include acute viral hepatitis, ischemic hepatitis, septic shock, toxin/medication, metabolic disorders, and so on (29). This patient was tested for viruses, heavy metals toxin, and genetic metabolic disease, all of which were negative. Since the spherical shape of the liver (detected by MRI, Figure 4) was susceptible to injury by vacuum sealing drainage, the increased intra-abdominal pressure may have led to hepatic ischemia. In this case, the ALT and AST returned to normal ranges after withdrawing VSD. Therefore, we think that the elevated ALT and AST may be associated with hepatic ischemia/reperfusion injury.

The mean small bowel length in a child with congenital short bowel is 57 cm (30). The patient’s small bowel length was only 50 cm, which was consistent with a congenital short bowel. It has been reported that the early onset of clinical signs, including such as vomiting and diarrhea, in children with congenital short bowel disease suggests a poor prognosis, and delayed presentation suggests a favorable outcome (12). This patient did not develop relevant symptoms early on, and full enteral feeding was eventually successfully established. The management goals for congenital short small bowel and short bowel syndrome caused by intestinal resection postoperatively are similar. During the early intestinal maladaptation period, parenteral nutrition support is required to gradually enhance intestinal adaptation and achieve enteral autonomy, while avoiding complications such as intestinal failure-related liver disease and catheter-related bloodstream infections (15, 31). Breast milk is the first choice for enteral nutrition for children with short bowel syndrome; however, when breast milk is not available, some scholars recommend the use of hydrolyzed or amino acid milk because children with short bowel syndrome are prone to cow’s milk protein allergy (32, 33). Breast milk was unavailable; therefore, we chose extensively hydrolyzed formulas (eHF) combined with active parenteral nutrition for early trophoblastic feeding. Owing to the low osmolality of eHF, its energy density (67–81 kcal/dL) could be increased with the use of additional milk powder. Moreover, eHF is more prone to inducing immune tolerance than the amino acid formula (34). After 19 days, she successfully transitioned to full enteral feeding by eliminating parenteral nutrition without complications such as intestinal failure-related liver disease or cow’s milk protein allergy.

## 4 Conclusion

A cohesive, multidisciplinary approach is recommended to combine surgical management with repair for a GO as early as possible; the approach can use VSD protective material as the initial protective shield while closely monitoring the intra-abdominal pressure to avoid abdominal compartment syndrome. Moreover, early enteral trophic feeding with an extensively hydrolyzed formula could be initiated for patients with a congenitally short small intestine when human milk is not available.

## Data availability statement

The original contributions presented in the study are included in the article/supplementary material, further inquiries can be directed to the corresponding author.

## Ethics statement

Written informed consent was obtained from the minor(s)’ legal guardian/next of kin for the publication of any potentially identifiable images or data included in this article.

## Author contributions

WZ: Data curation, Investigation, Validation, Visualization, Writing – original draft. YW: Conceptualization, Methodology, Writing – review & editing. CP: Formal analysis, Investigation, Methodology, Supervision, Writing – review & editing. XZ: Data curation, Formal analysis, Methodology, Writing – review & editing. HY: Conceptualization, Data curation, Formal analysis, Resources, Writing – review & editing. LZ: Conceptualization, Data curation, Formal analysis, Funding acquisition, Investigation, Methodology, Project administration, Resources, Supervision, Writing – original draft, Writing – review & editing, Software, Validation, Visualization.
